# Absolute nutrient concentration measurements in cell culture media: ^1^H *q*-NMR spectra and data to compare the efficiency of pH-controlled protein precipitation versus CPMG or post-processing filtering approaches

**DOI:** 10.1016/j.dib.2016.05.054

**Published:** 2016-05-30

**Authors:** Luca Goldoni, Tiziana Beringhelli, Walter Rocchia, Natalia Realini, Daniele Piomelli

**Affiliations:** a Drug Discovery and Development, Istituto Italiano di Tecnologia, via Morego 30, 16163 Genova, Italy; b Department of Chemistry, University of Milan, via Golgi 19, 20133 Milano, Italy; c CONCEPT Lab, Istituto Italiano di Tecnologia, via Morego 30, 16163 Genova, Italy; d Departments of Anatomy and Neurobiology, Pharmacology and Biological Chemistry, University of California, Irvine, CA 92697, USA

**Keywords:** ^1^H NMR, pH-controlled serum removal, PULCON, Accuracy, CPMG, Deconvolution

## Abstract

The NMR spectra and data reported in this article refer to the research article titled “A simple and accurate protocol for absolute polar metabolite quantification in cell cultures using *q*-NMR” [1]. We provide the ^1^H *q*-NMR spectra of cell culture media (DMEM) after removal of serum proteins, which show the different efficiency of various precipitating solvents, the solvent/DMEM ratios, and pH of the solution. We compare the data of the absolute nutrient concentrations, measured by PULCON external standard method, before and after precipitation of serum proteins and those obtained using CPMG (Carr-Purcell-Meiboom-Gill) sequence or applying post-processing filtering algorithms to remove, from the ^1^H *q*-NMR spectra, the proteins signal contribution. For each of these approaches, the percent error in the absolute value of every measurement for all the nutrients is also plotted as accuracy assessment.

**Specifications Table**

**Table:** 

Subject area	*Biology*
More specific subject area	*Absolute nutrient quantification in cell culture medium by Nuclear Magnetic Resonance (NMR)*
Type of data	*Spectra, tables and graphs*
How data was acquired	^*1*^*H q-NMR (quantitative NMR) using PULCON method for the absolute measurements of nutrients*
Data format	*Raw and analyzed*
Experimental factors	*All the samples were freeze-dried and then reconstituted in the deuterated buffer before*^*1*^*H q-NMR analysis*
Experimental features	*All the NMR spectra were acquired on a Bruker AvanceIII 600 MHz spectrometer equipped with 5 mm QCI cryoprobe*
Data source location	*IIT (Istituto Italiano di Tecnologia), Genova, Italy*
Data accessibility	*Data are available with this article*

**Value of the data**

• NMR spectra reveal that the effectiveness of methanol at precipitating proteins is strongly pH-dependent.
• The data provide a comprehensive comparison among different strategies aimed at overcoming the matrix interference of proteins in absolute nutrient quantification by ^1^H *q*-NMR.
• The data concerning the accuracy degradation for all nutrients at every concentration may help to choose the most suitable approach for protein removal.

## 1 Data

**Fig. 1:**
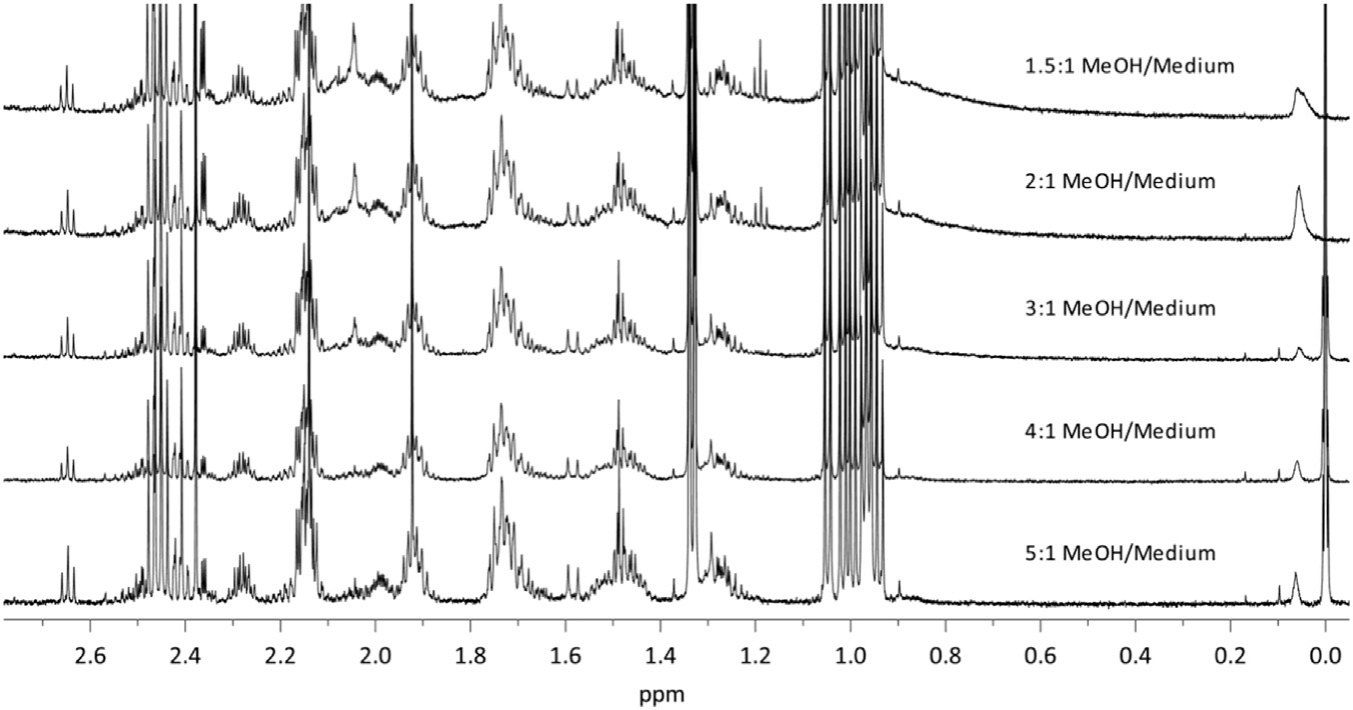
^1^H *q*-NMR spectra of a 1× DMEM solution, containing 10% of serum, after serum protein precipitation with different ratio of MeOH, freeze-drying and reconstitution in deuterated buffer.

**Fig. 2:**
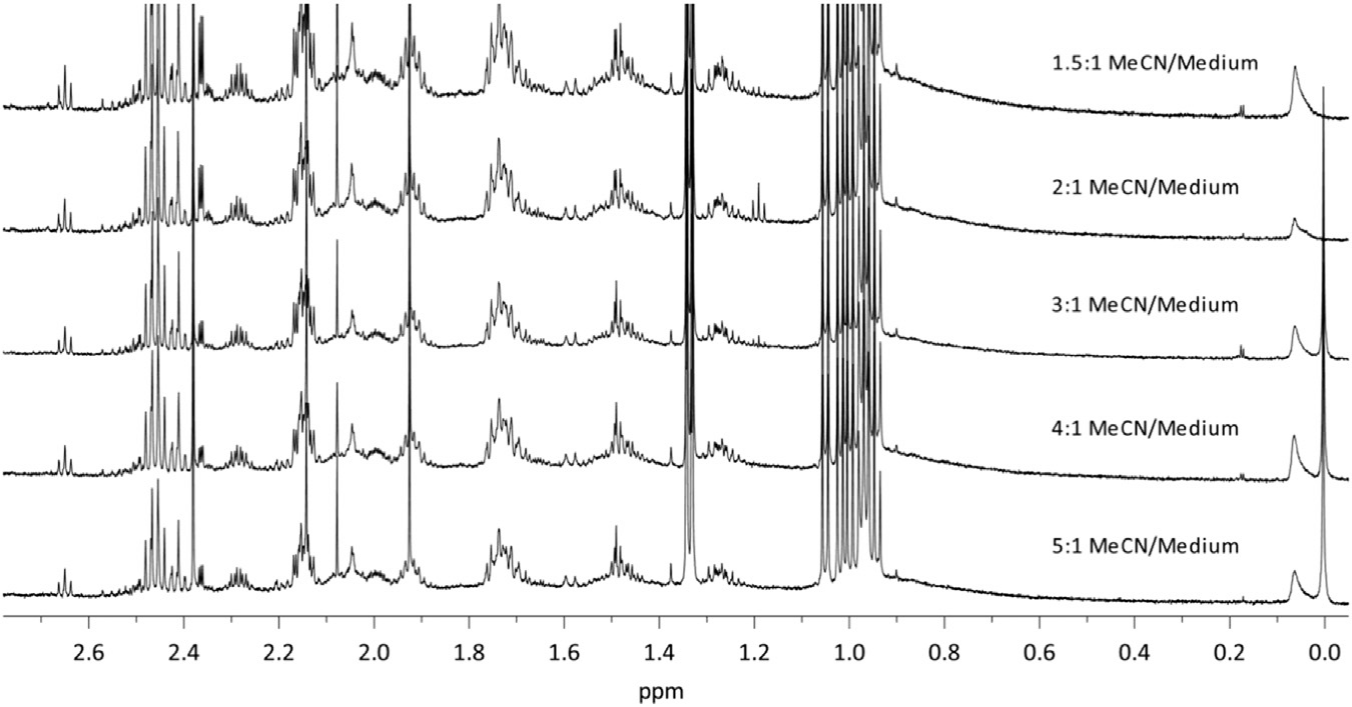
^1^H *q*-NMR spectra of a 1× DMEM solution, containing 10% serum, after serum protein precipitation with different ratio of MeCN, freeze-drying and reconstitution in deuterated buffer.

**Fig. 3:**
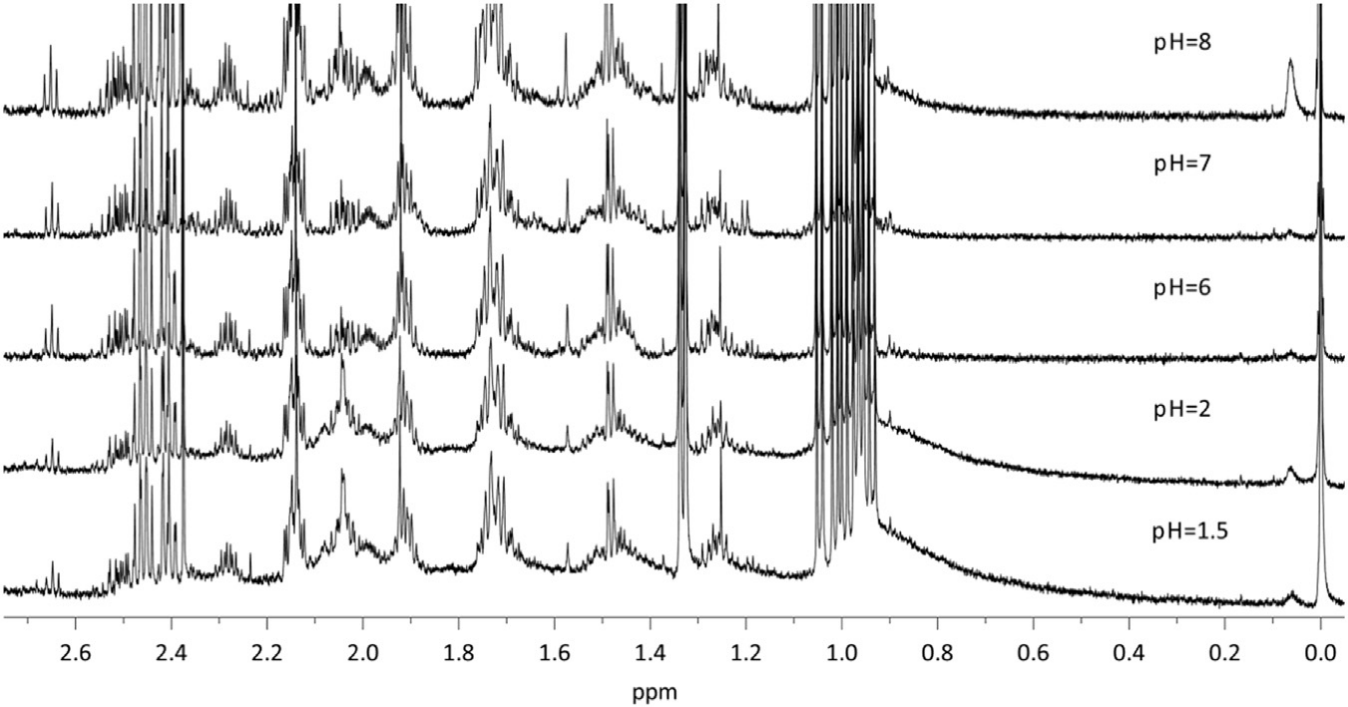
pH dependence of protein precipitation from a 1× DMEM solution, containing 10% serum (methanol:DMEM-serum, 3:1). ^1^H *q*-NMR spectra obtained after freeze-drying and reconstitution in deuterated buffer.

**Fig. 4:**
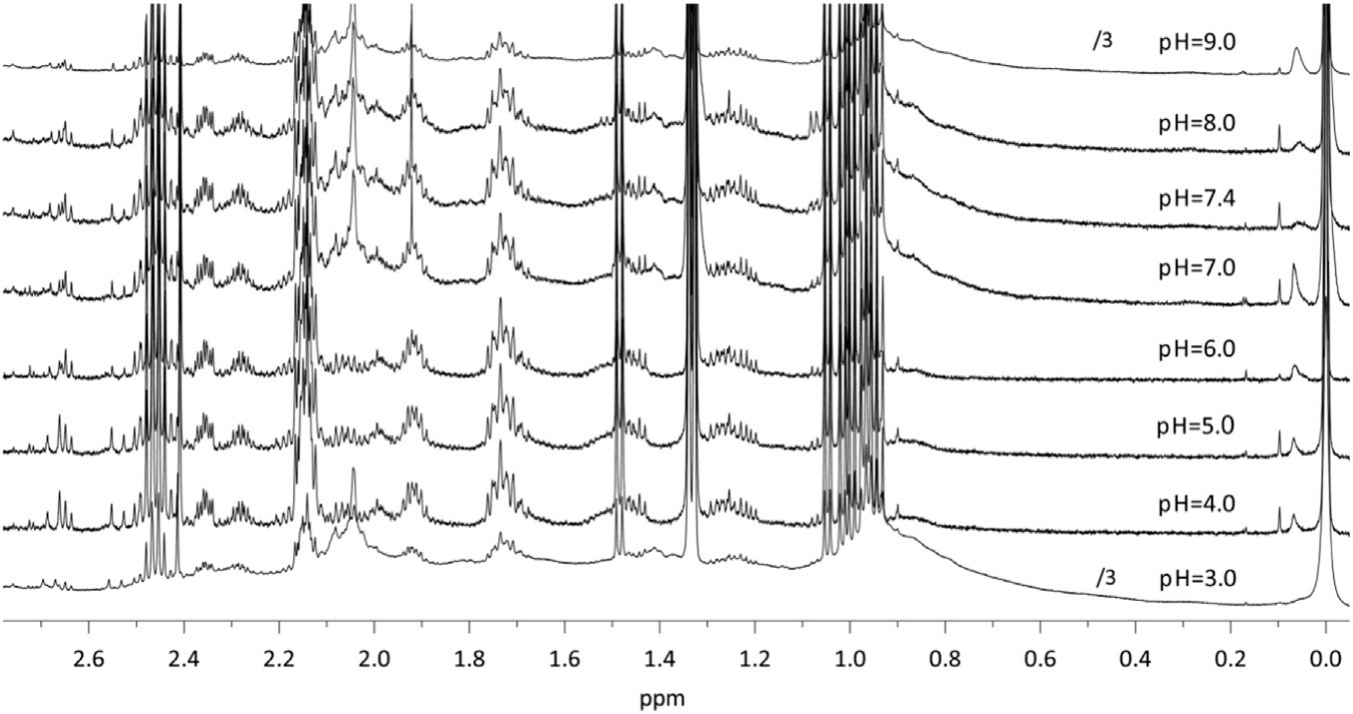
pH dependence of protein precipitation from a 1× DMEM solution, containing 80% serum (methanol:DMEM-serum, 3:1). ^1^H *q*-NMR spectra obtained after freeze-drying and reconstitution in deuterated buffer.

**Fig. 5:**
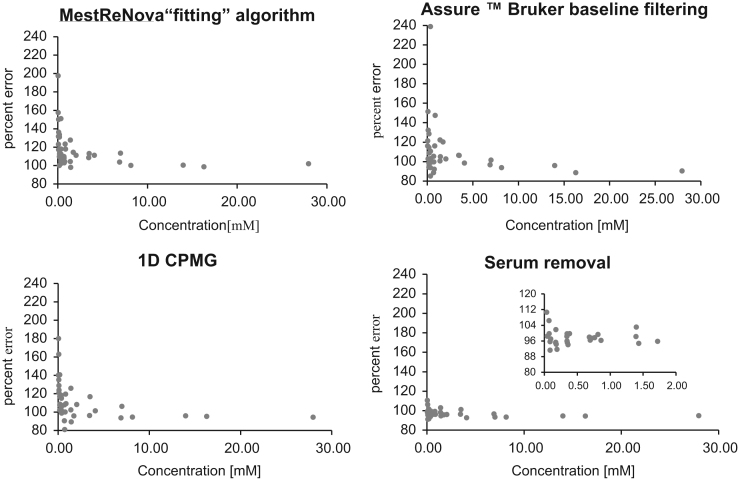
Percent error versus analyte concentration, as assessed using each of the four tested approaches for the removal of protein serum contribution.

Dataset provided in this article shows selected regions of the ^1^H *q*-NMR (quantitative NMR) spectra after protein serum precipitation (Fig. 1, Fig. 2) using variable ratios of different solvents (Table 1). ^1^H *q-*NMR spectra reported in Fig. 3, Fig. 4 show the pH dependence of serum protein precipitation from medium containing 10% and 80% of serum, respectively. We also provide absolute nutrient concentrations for each nutrient (Table 2) and the corresponding percent errors obtained with each of the four evaluated approaches (Fig. 5).

## 2 Experimental design, materials and methods

### 2.1 DMEM model solutions

Dulbecco׳s Modified Eagle׳s Medium solutions were prepared by serial dilutions of a standard stock 2× DMEM freshly made from powder dissolved in MilliQ water. Model solutions with serum were prepared by addition of bovine serum (10% or 80%) to DMEM solutions.

### 2.2 Freeze-drying

0.4 mL of medium (DMEM, DMEM-serum) were transferred to a 15 mL Falcon tube. Solutions were frozen in liquid N_2_ and freeze-dried at least for 3 h, till the formation of a fluffy solid. The powder was reconstituted in deuterated buffer for NMR analysis (100 mM potassium phosphate/D_2_O buffer, pH=7.15, containing TSP as reference).

### 2.3 Removal of serum proteins

Various conditions for serum protein removal were tested (Table 1). The following general scheme was applied: an appropriate volume of ice-cold precipitation solvent was added to 0.4 mL of DMEM-serum on ice, the solution was stirred for 20 s, incubated for 20 min and centrifuged at 2100 rcf (relative centrifugal force) for 15 min at 4 °C. The supernatants were collected, diluted with 3.4 mL water, freeze-dried for at least 12 h, till the formation of a fluffy solid and reconstituted in 0.4 mL of deuterated buffer for analysis. DMEM-serum solutions were acidified by adding (TFA).

### 2.4 NMR spectroscopy

NMR experiments were performed, without spinning, on a Bruker AvanceIII 600 MHz spectrometer equipped with 5 mm QCI cryoprobe with z shielded pulsed-field gradient coil. Before each acquisition, automatic matching and tuning were run, the 90° pulse was optimized by means of an automatic pulse calculation routine [2] and the homogeneity automatically adjusted on each sample-tube. Before data acquisition the samples were equilibrated for 2 min inside the probe and the temperature was actively controlled at 298 K. 32 transients were accumulated, at a fixed receiver gain, using 64 K complex data points, over a spectral width of 20.6 ppm and with a relaxation delay of 30 s. An inter pulse spacing of 2.3 ms and a duty cycles of 20 were adopted for 1D CPMG. An exponential line-broadening (0.1 Hz) was applied to FIDs (Free Induction Decay) before Fourier transform. The spectra were manually phased and automatically baseline corrected (as preferred in metabolomics, Bharti et al. [3]).

### 2.5 Concentration measurements

Nutrient concentrations, using PULCON (PUlse Length Based Concentration Determination) procedure [4], [5], [6], [7], were measured on the DMEM model solutions with serum: (i) without protein removal, directly on the rough freeze-dried/reconstituted solutions (ii) after pH-controlled serum protein removal [1], (iii) after applying post-processing filtering procedures [1] (Assure™ Bruker and MestReNova software tools) and (iv) using the CPMG acquisition scheme [1], (Table 2). The signal decay due to its intrinsic T_2_ was taken into account to correlate the signal intensities measured in the 1D CPMG experiments to the nutrient concentrations.

## Figures and Tables

**Table 1:** Experimental conditions tested for serum removal.

MeOH:DMEM	MeCN:DMEM	MeCN:DMEM (+TFA)	MeOH:DMEM (+TFA)
**1.5:1**	1.5:1	1.5:1	1.5:1
**2:1**	2:1	2:1	2:1
**3:1**	3:1	3:1	3:1
**5:1**	5:1	5:1	5:1

**Table 2:** Theoretical and measured nutrient absolute concentrations obtained by means of different methods for the removal of protein serum contribution (average value of three analytical replicates).

**DMEM solutions**	**Theoretical conc.**	**Measured conc.**	Theoretical conc. after the serum addition (*)	**Measured conc. after the serum addition**	**Measured conc. after serum removal method**	**Measured conc. after serum removal, (independent solutions)**	**Measured conc. Assure ^TM^ Bruker**	**Measured conc. MestReNova “fitting” algorithm**	**Measured conc. 1D-CPMG, after the T_2_ decay correction**
**α-Glucose**									
**2×**	17.9152	17.9152	16.2865	16.1337	15.3976	15.4296	14.4357	16.0800	15.5293
**1×**	8.9576	8.9430	8.1433	8.1397	7.6132	7.3983	7.6373	8.1600	7.7039
**0.5×**	4.4788	4.4347	4.0716	4.1688	3.7776	3.8590	4.0110	4.5276	4.1294
**0.25×**	2.2394	2.2119	2.0358	2.2378	1.9575	1.9356	2.0919	2.2633	2.2045
**0.1×**	0.8958	0.9097	0.8143	0.9554	0.8987	0.8992	0.9457	1.0050	0.9740

**β-Glucose**									
**2×**	30.7454	30.7454	27.9504	27.5983	26.5280	26.7599	25.2684	28.5155	26.4012
**1×**	15.3727	15.3889	13.9752	13.4447	13.2250	12.8986	13.4082	14.0419	13.4126
**0.5×**	7.6864	7.5570	6.8976	6.9238	6.5333	6.6089	7.0970	7.9320	7.4270
**0.25×**	3.8432	3.7078	3.4938	3.7448	3.5377	3.3781	3.7157	3.9422	4.0806
**0.1×**	1.5373	1.5684	1.3975	1.7402	1.4403	1.5690	1.7088	1.7850	1.7606

**Histidine**									
**2×**	0.3742	0.3777	0.3434	0.3908	0.3294	0.3344	0.2923	0.3508	0.3506
**1×**	0.1871	0.1848	0.1717	0.2351	0.1636	0.1614	0.1616	0.1719	0.2009
**0.5×**	0.0936	0.0899	0.0858	0.0781	0.0783	0.0850	0.0883	0.1007	0.1107
**0.25×**	0.0468	0.0464	0.0429	0.0792	0.0422	0.0463	0.0521	0.0667	0.0773
**0.1×**	0.0234		0.0172						

**Phenylalanine**									
**2×**	0.8398	0.8398	0.7635	0.8838	0.7456	0.7248	0.7037	0.7970	0.7646
**1×**	0.4199	0.4248	0.3817	0.4968	0.3807	0.3751	0.3787	0.4048	0.3776
**0.5×**	0.2100	0.2071	0.1909	0.2791	0.1750	0.1856	0.2077	0.2151	0.2073
**0.25×**	0.1050	0.1040	0.0954	0.1472	0.0926	0.0926	0.1263	0.1301	0.1183
**0.1×**	0.0420		0.0382	0.8838					

**Tyrosine**									
**2×**	0.7516	0.7516	0.6833	0.7927	0.6692	0.6485	0.6385	0.7507	0.6188
**1×**	0.3758	0.3715	0.3416	0.4968	0.3359	0.3321	0.3482	0.3513	0.3144
**0.5×**	0.1879	0.1894	0.1708	0.2353	0.1622	0.1594	0.1934	0.1875	0.1745
**0.25×**	0.0940	0.0868	0.0854	0.1143	0.0817	0.0811	0.1133	0.1282	0.1034
**0.1×**	0.0376	0.0338	0.0342	0.0834	0.0378	0.0337	0.0592	0.0675	0.0479

**Glutamine**									
**2×**	7.5753	7.5753	6.8866	8.0064	6.6608	6.5342	6.6593	7.1498	6.4549
**1×**	3.7877	3.8009	3.4433	4.3168	3.3191	3.2062	3.6671	3.7409	3.3151
**0.5×**	1.8938	1.9008	1.7217	2.2081	1.6492	1.6651	2.0699	1.9709	1.6511
**0.25×**	0.9469	0.9307	0.8608	1.0557	0.8288	0.8277	1.2699	1.0153	0.9400
**0.1×**	0.3788	0.3932	0.3443	0.4242	0.3432	0.3246	0.8226	0.5200	0.3638

**Isoleucine**									
**2×**	1.5782	1.5782	1.4347	1.6679	1.3593	1.3624	1.5074	1.4074	1.2822
**1×**	0.7891	0.7813	0.7174	0.9557	0.6987	0.6640	0.7153	0.7391	0.5812
**0.5×**	0.3946	0.3901	0.3587	0.4930	0.3374	0.3361	0.3698	0.4234	0.4130
**0.25×**	0.1973	0.1936	0.1793	0.2219	0.1691	0.1760	0.2306	0.2349	0.1929
**0.1×**	0.0789	0.0770	0.0717	0.0976	0.0715	0.0712	0.1087	0.0884	0.0970

**Valine**									
**2×**	1.5634	1.5318	1.3925	1.6389	1.3681	1.3491	1.4019	1.4541	1.4270
**1×**	0.7817	0.7690	0.6963	0.8822	0.6711	0.6721	0.7344	0.7540	0.7517
**0.5×**	0.3909	0.3769	0.3481	0.4207	0.3312	0.3310	0.3852	0.3917	0.4116
**0.25×**	0.1954	0.1857	0.1741	0.2044	0.1771	0.1799	0.1994	0.2325	0.2449
**0.1×**	0.0782	0.0740	0.0696	0.0841	0.0741	0.0822	0.0205	0.0917	0.1135

^(*)^Considering the dilution due to the serum addition (10% volume).
