# Supplementary material for: Absolute nutrient concentration measurements in cell culture media: 1H q-NMR spectra and data to compare the efficiency of pH-controlled protein precipitation versus CPMG or post-processing filtering approaches
Source: Data Brief. 2016 May 30;8:387–93. doi: 10.1016/j.dib.2016.05.054 (PMC4909824; doi:10.1016/j.dib.2016.05.054)

Conflicts of Interest Statement

Ref: Ms. No.: DIB-D-16-00119

Manuscript title:

Absolute nutrient concentration measurements in cell culture media: 1H q-NMR spectra and data to compare the efficiency of pH-controlled protein precipitation versus CPMG or post processing filtering approaches.

The authors whose names are listed immediately below certify that they have no affiliations with or involvement in any organization or entity with any financial interest (such as honoraria; educational grants; participation in speakers' bureaus; membership, employment, consultancies, stock ownership, or other equity interest; and expert testimony or patent-licensing arrangements), or non-financial interest (such as personal or professional relationships, affiliations, knowledge or beliefs) in the subject matter or materials discussed in this manuscript.

Author names:

Luca Goldoni,[‡*] Tiziana Beringhelli[//] , Walter Rocchia[``], Natalia Realini[‡], Daniele Piomelli[‡,†]

Affiliations:

[‡]*Drug Discovery and Development, Istituto Italiano di Tecnologia, via Morego 30, 16163 Genova, Italy*

[//] *Department of Chemistry, University of Milan, via Golgi 19, 20133 Milano, Italy*

[``]*CONCEPT Lab, Istituto Italiano di Tecnologia, via Morego 30, 16163 Genova*

[†]*Departments of Anatomy and Neurobiology, Pharmacology and Biological Chemistry, University of California, Irvine, CA 92697, USA*

Monday, April 18, 2016

Luca Goldoni, for all the authors

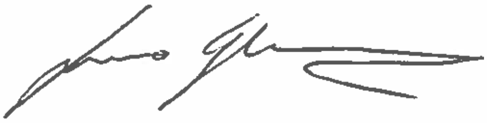

Supplement: Supplementary file 1 — Supplementary material [file mmc1.pdf]
