# Supplementary material for: Absolute nutrient concentration measurements in cell culture media: 1H q-NMR spectra and data to compare the efficiency of pH-controlled protein precipitation versus CPMG or post-processing filtering approaches
Source: Data Brief. 2016 May 30;8:387–93. doi: 10.1016/j.dib.2016.05.054 (PMC4909824; doi:10.1016/j.dib.2016.05.054)
Supplement: Supplementary file 2 — Supplementary material [file mmc2.zip › Raw data (FID)/Detailed list of contents.docx]

**Detailed list of contents**

The file name of FID (free induction decay) and a brief description of the content are herein enclosed.

**Figure 1**

The FID of spectra obtained after serum protein precipitation, using methanol (MeOH):

| **File Name** | **Description** |
| --- | --- |
| MeOH_1.5_1 | MeOH:DMEM_serum, 1.5:1 |
| MeOH_2_1 | MeOH:DMEM_serum, 2:1 |
| MeOH_3_1 | MeOH:DMEM_serum, 3:1 |
| MeOH_4_1 | MeOH:DMEM_serum, 4:1 |
| MeOH_5_1 | MeOH:DMEM_serum, 5:1 |

**Figure 2**

The FID of spectra obtained after serum protein precipitation, using acetonitrile (MeCN):

| **File Name** | **Description** |
| --- | --- |
| MeCN_1.5_1 | MeCN:DMEM_serum, 1.5:1 |
| MeCN_2_1 | MeCN:DMEM_serum, 2:1 |
| MeCN_3_1 | MeCN:DMEM_serum, 3:1 |
| MeCN_4_1 | MeCN:DMEM_serum, 4:1 |
| MeCN_5_1 | MeCN:DMEM_serum, 5:1 |

**Figure 3**

The FID of spectra obtained after serum protein precipitation, using methanol (MeOH:DMEM-serum, 3:1), at different pH.

| **File Name** | **Description** |
| --- | --- |
| 10pH_1.5 | (10% of serum) at pH = 1.5 |
| 10pH_2 | (10% of serum) at pH = 2 |
| 10pH_6 | (10% of serum) at pH = 6 |
| 10pH_7 | (10% of serum) at pH = 7 |
| 10pH_8 | (10% of serum) at pH = 8 |

**Figure 4**

The FID of spectra obtained after serum protein precipitation, using methanol (MeOH:DMEM-serum, 3:1), at different pH:

| **File Name** | **Description** |
| --- | --- |
| 80pH_3 | (80% of serum) at pH = 3 |
| 80pH_4 | (80% of serum) at pH = 4 |
| 80pH_5 | (80% of serum) at pH = 5 |
| 80pH_6 | (80% of serum) at pH = 6 |
| 80pH_7 | (80% of serum) at pH = 7 |
| 80pH_7.4 | 80% of serum) at pH = 7.4 |
| 80pH_8 | (80% of serum) at pH = 8 |
| 80pH_9 | (80% of serum) at pH = 9 |
